# Moderators of the Impact of (Poly)Phenols Interventions on Psychomotor Functions and BDNF: Insights from Subgroup Analysis and Meta-Regression

**DOI:** 10.3390/nu12092872

**Published:** 2020-09-19

**Authors:** Achraf Ammar, Khaled Trabelsi, Omar Boukhris, Bassem Bouaziz, Patrick Müller, Jordan M. Glenn, Karim Chamari, Notger Müller, Hamdi Chtourou, Tarak Driss, Anita Hökelmann

**Affiliations:** 1 Institute of Sport Sciences, Otto-von-Guericke University, 39104 Magdeburg, Germany; anita.hoekelmann@ovgu.de; 2 High Institute of Sport and Physical Education, University of Sfax, Sfax 3000, Tunisia; trabelsikhaled@gmail.com (K.T.); omarboukhris24@yahoo.com (O.B.); h_chtourou@yahoo.fr (H.C.); 3 Research Laboratory: Education, Motricité, Sport et Santé, EM2S, LR19JS01, High Institute of Sport and Physical Education of Sfax, University of Sfax, Sfax 3000, Tunisia; 4 Activité Physique, Sport et Santé, UR18JS01, Observatoire National du Sport, Tunis 1003, Tunisia; 5 Higher Institute of Computer Science and Multimedia of Sfax, University of Sfax, Sfax 3000, Tunisia; bassem.bouaziz@isims.usf.tn; 6 German Center for Neurodegenerative Diseases (DZNE), 39104 Magdeburg, Germany; patrick.mueller@dzne.de (P.M.); notger.mueller@dzne.de (N.M.); 7 Department of Neurology, Medical Faculty, Otto von Guericke University, 39104 Magdeburg, Germany; 8 Department of Health, Exercise Science Research Center, Human Performance and Recreation, University of Arkansas, Fayetteville, AR 72701, USA; jordan@neurotrack.com; 9 Neurotrack Technologies, 399 Bradford St, Redwood City, CA 94063, USA; 10 ASPETAR, Qatar Orthopaedic and Sports Medicine Hospital, Doha PoBox 29222, Qatar; karim.chamari@aspetar.com; 11 Laboratory “Sport Performance Optimization”, (CNMSS), ISSEP Ksar-Said, Manouba University, Manouba 1004, Tunisia; 12 Interdisciplinary Laboratory in Neurosciences, Physiology and Psychology: Physical Activity, Health and Learning (LINP2-2APS), UFR STAPS, UPL, Paris Nanterre University, 92000 Nanterre, France; tarak.driss@parisnanterre.fr

**Keywords:** antioxidant, aging, psychomotor functions, brain plasticity, brain functions, cognition

## Abstract

Background: Recent anti-aging interventions have shown contradictory impacts of (poly)phenols regarding the prevention of cognitive decline and maintenance of brain function. These discrepancies have been linked to between-study differences in supplementation protocols. This subgroup analysis and meta-regression aimed to (i) examine differential effects of moderator variables related to participant characteristics and supplementation protocols and (ii) identify practical recommendations to design effective (poly)phenol supplementation protocols for future anti-aging interventions. Methods: Multiple electronic databases (Web of Science; PubMed) searched for relevant intervention published from inception to July 2019. Using the PICOS criteria, a total of 4303 records were screened. Only high-quality studies (*n* = 15) were included in the final analyses. Random-effects meta-analysis was used, and we calculated standard differences in means (SDM), effect size (ES), and 95% confidence intervals (CI) for two sufficiently comparable items (i.e., psychomotor function and brain-derived neurotrophic factor (BDNF)). When significant heterogeneity was computed (*I*^2^ > 50%), a subgroup and meta-regression analysis were performed to examine the moderation effects of participant characteristics and supplementation protocols. Results: The reviewed studies support the beneficial effect of (poly)phenols-rich supplementation on psychomotor functions (ES = −0.677, *p* = 0.001) and brain plasticity (ES = 1.168, *p* = 0.028). Subgroup analysis revealed higher beneficial impacts of (poly)phenols (i) in younger populations compared to older (SDM = −0.89 vs. −0.47 for psychomotor performance, and 2.41 vs. 0.07 for BDNF, respectively), (ii) following an acute compared to chronic supplementation (SDM = −1.02 vs. −0.43 for psychomotor performance), and (iii) using a phenolic compound with medium compared to low bioavailability rates (SDM = −0.76 vs. −0.68 for psychomotor performance and 3.57 vs. 0.07 for DBNF, respectively). Meta-regressions revealed greater improvement in BDNF levels with lower percentages of female participants (Q = 40.15, df = 6, *p* < 0.001) and a skewed scatter plot toward a greater impact using higher (poly)phenols doses. Conclusion: This review suggests that age group, gender, the used phenolic compounds, their human bioavailability rate, and the supplementation dose as the primary moderator variables relating to the beneficial effects of (poly)phenol consumption on cognitive and brain function in humans. Therefore, it seems more advantageous to start anti-aging (poly)phenol interventions in adults earlier in life using medium (≈500 mg) to high doses (≈1000 mg) of phenolic compounds, with at least medium bioavailability rate (≥9%).

## 1. Introduction

Aging, a complex biological process, is inescapably connected with age-related health decline, affecting several aspects of cognitive functioning [1]. A primary factor associated with age-related cognitive decline is the development of neuroinflammation [2]. This nervous tissue inflammation may be initiated by a variety of cues, such as traumatic brain injury [3] infection, autoimmunity [4] toxic metabolites, or a disequilibrium redox state in favor of prooxidants (e.g., reactive oxygen species (ROS)) [5,6]. Regarding this, increased lipid peroxidation and protein oxidation in aged hippocampus and the cerebral cortex have been shown to increase the susceptibility of neurons to apoptosis via the generated reactive protein oxidation and lipid peroxidation products (e.g., 4-hydroxy-2-nonenal) [6,7]. In a state of chronic oxidative stress, ROS-induced intracellular signaling pathways are altered, leading to dysregulation of the inflammatory response [8]. This loss in the regulation of signal transduction by the cells is accompanied by an increased production of damage-associated molecular patterns and an increased secretion of proinflammatory molecules, which act together to promote neuroinflammation and may play an important role in neuron dysfunction and ultimately cognitive decline [2,8,9].

Given the decreased activity of many endogenous antioxidant enzymes, such as superoxide dismutase, glutathione peroxidase, and catalase in aged hippocampus and the cerebral cortex [10,11], a huge array of existing literature has highlighted the importance of exogenous antioxidants as potential anti-aging agents [12,13,14,15,16]. The most widely known exogenous antioxidants are carotenoids (lycopene, lutein, zeaxanthin, α- and β-carotene, β-cryptoxanthin), vitamin E (α- and γ-tocopherol), vitamin C, and (poly)phenols [17], with the latter exhibiting the highest antioxidant capabilities [18]. Considering the increased effectiveness of (poly)phenols in counteracting age-related oxidative stress, recent human studies have examined the role of natural (poly)phenols-rich products in the prevention of cognitive decline and maintenance of brain function [15,16]. Several studies have shown that the consumption of (poly)phenols-rich supplementation can benefit cognitive decline in older adults [19,20,21], as well as in young- and middle-aged populations [22,23,24]. Additionally, polyphenols bind to nuclear estrogen receptor α (ERα) and β (ERβ), thus inducing neuroprotective effects. These effects are particularly noticeable in human cells, where they mimic or inhibit actions of endogenous estrogens [25]. Contrarywise, other large-scale clinical trials investigating similar populations have failed to produce similar results [26,27,28,29].

Discrepancies between these findings have been suggested to be linked to the between-studies differences in supplementation protocols, amongst others [15]. Specifically, a recent systematic review and meta-analysis by our research group underlined the specificity of phenolic compounds, (poly)phenol dose, and bioavailability as the primary determinants of the efficacy of (poly)phenols-rich supplementation in anti-aging interventions [15,16]. The results of this publication suggested that an intermediate dose of (poly)phenols with intermediate to high human rates of bioavailability are necessary in order for them to successfully cross the blood–brain barrier, exerting significant effects on cognitive function and brain health [15].

Furthermore, the application of anti-aging interventions to young, healthy individuals has theoretical advantages to reduce the onset of brain-related aging processes [30,31]. Our research group additionally underlined age as an important moderator in (poly)phenol efficacy in anti-aging interventions [16]. Another recent systematic review focusing on young- and middle-aged populations suggested that only a low- to medium-dose of phenolic components is necessary at younger ages to elicit promising effects on brain health [16].

Although the aforementioned suggestions in our previous reports [15,16] are of importance to aid in designing future anti-aging (poly)phenol interventions, the suggested phenolic doses, bioavailability rate, and target age group eventually need to be confirmed by a subgroup meta-analysis of comparable data from age-group studies. Moreover, given the large range of phenolic compounds (i.e., flavanols, anthocyanidins, flavones, isoflavones, flavonols, and flavanones/flavanonols) and their natural fruits and vegetables sources (e.g., celery, onions, oregano herbs, broccoli, green tea, dark chocolate, red wine, soy, citrus fruit, leeks, berry fruits, and parsley) [13,32], it is also important to identify the most effective phenolic compounds during anti-aging preventive interventions.

Finally, there is extensive literature documenting the moderating effects of gender on cognition and brain aging process (i.e., rate of blood flow, pattern of glucose metabolism, and receptors activity), with some evidence suggesting that women show less age-associated cognitive decline, while men undergo more progressive decreases in frontotemporal brain volume [33]. Therefore, performing a gender subgroup analysis of data from anti-aging (poly)phenol intervention is warranted.

To overcome the gaps in recently published meta-analyses, this review was designed to (i) examine differential effects of moderator variables related to participants’ characteristics (i.e., gender/age) and the supplementation protocol (i.e., nature, dose, phenolic compound’s bioavailability, and the duration of the intervention period) and (ii) identify practical recommendations to design effective (poly)phenol supplementation protocols for future anti-aging interventions based on optimal phenolics’ compounds, dose, bioavailability rate, optimal intervention period, and optimal target population.

## 2. Materials and Methods

The Preferred Reporting Items for Systematic Reviews and Meta-Analysis (PRISMA) guidelines was followed in the present systematic review [34].

### 2.1. Search Stratgies and Sources of Data

Up to July 2019, an electronic comprehensive systematic search was performed using electronic databases (PubMed and Web of Science), without applying any time limits. The search was limited to only English publications. The search strategy as well as the search terms were similar to the ones utilized by Ammar et al. [15,16]. Additionally, in order to minimize the risk of missing relevant publications, the reference lists of included manuscripts and similar journal citations identified from Google Scholar were reviewed. Two independent researchers considered each of the articles for their inclusion appropriateness. A discussion with a third researcher was performed in cases of uncertainty to determine the final inclusion or exclusion of the paper. Further information on the search process and inclusion and exclusion criteria are presented in Table 1.

### 2.2. Study Selection

**Figure 1:**
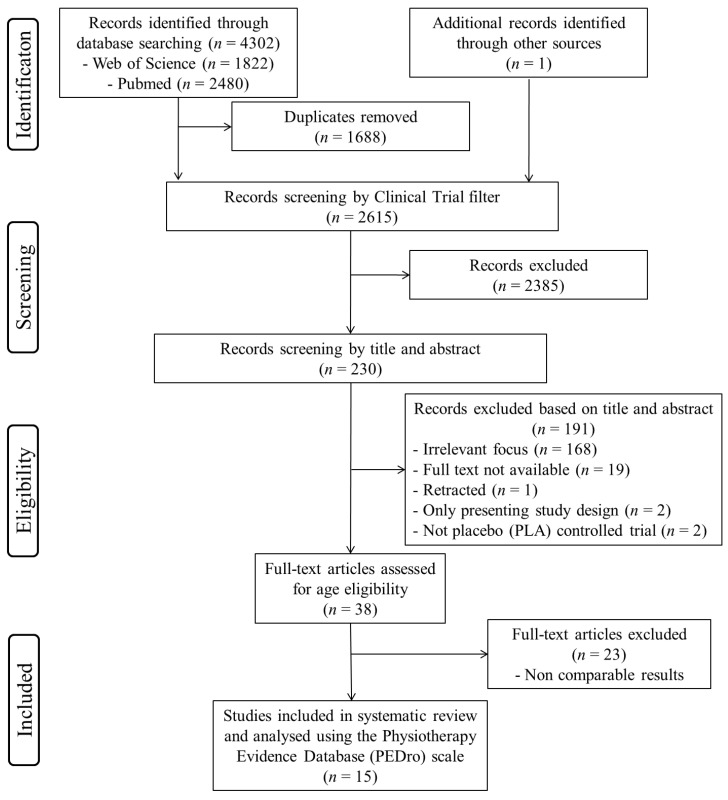
Flow diagram of the literature selection process.

The process used for selecting articles is outlined in Figure 1. EndNote X8 (produced by Clarivate Analytics, Philadelphia, USA) was used to remove duplicate articles in the initial search results. Following the removal of duplicates, the titles and abstracts of all unique hits were screened by two authors for eligibility, and disagreements were resolved by consensus. Then, inclusion and exclusion of the article list was conducted via full text screening in accordance to the Participants, Intervention, Comparative, Outcome and Study design (PICOS); inclusion and exclusion criteria are detailed in Table 1. Reasons for article exclusion were recorded during this process.

### 2.3. Data Collection

Using a pilot-tested extraction form, two authors independently collected data, and disagreements were resolved via mutual consensus. Data that were extracted included (i) characteristics of participants (i.e., sex, age, participant numbers), (ii) supplementation protocol (i.e., nature, dose and bioavailability of the used phenolic compounds, and the duration of the intervention period), and (iii) key outcomes from anti-aging based-(poly)phenols intervention on psychomotor performance (i.e., evaluated during Trail Making Test (TMTa) and Reaction Time Test (RTT)) and brain plasticity (i.e., evaluated using the brain-derived neurotrophic factor (BDNF) measurements).

TMT: Traditionally, trail making tests reflect a multiple of cognitive processes such as shifting, attention, sequencing, visual search and scanning, psychomotor speed, flexibility, abstraction, ability to execute and adjust an action plan, and the capability to sustain two simultaneous trains of thought [30]. TMTa includes 25 numbered circles (1–25) distributed throughout a sheet of paper., Then, the patient draws lines connecting each number in ascending order, as quickly as possible, without ever lifting the writing instrument [35].

RTT Example: A white square appears 30 times, at random intervals, in a computer screen’s center. A “yes” button is pressed as soon as the stimulus is visible [23].

TMTa and RTT scores are calculated as the time required to complete the task in seconds; higher scores indicate increased levels of impairment [20,35].

### 2.4. Quality Assessment

Methodological study quality was assessed via the Physiotherapy Evidence Database (PEDro) scale [15,16,36]. PEDro is a reliable and objective tool based on the Delphi list developed by Verhagen et al. [37]. To identify which randomized controlled trials are externally (criteria 1) and internally (criteria 2–9) valid with interpretable results, each paper was independently assessed twice by two independent authors using the 11-item checklist to yield a maximum score of 10 (the sum of awarded points for criteria 2–11). Points are only awarded when a criterion is clearly satisfied. A score of 9–10 on the PEDro scale were considered to be of “high quality”, scores of 5–8 were considered to be of “moderate quality”, and studies that scored below 5 were considered to be of “low quality” [15,16,38].

### 2.5. Statistical Analysis

The software “Comprehensive Meta-Analysis” (CMA for Windows, version 3, Biostat, Englewood, NJ, USA) was used for the meta-analysis (MA). Given the level of cognitive task variability and brain measurement techniques between included studies, only psychomotor performance (TMTa or RTT) and BDNF were sufficiently comparable and thus included in this MA. To calculate effect size, performance in psychomotor tests was recorded in seconds (s), and BDNF concentrations were collated using ng/mL. For those studies where net changes in the groups were not directly reported, effect size (ES) was computed through subtraction of the values at the intervention endpoint from baseline. Calculations of standard deviations of mean differences were completed via (SD = square root ((SD pre-treatment)^2^ + (SD post-treatment)^2^ − (2R × SD pre-treatment × SD post-treatment))); the correlation coefficient (R) was assumed to be 0.5 [39,40]. ESs as well as their 95% confidence intervals (CI) were calculated via Cohen’s method, reflecting the standardized difference in means (SDM) between measured parameters (i.e., psychomotor performance and BDNF), in response to (poly)phenols-rich supplementation and placebo. ES were determined to be trivial (ES < 0.2), small (ES of 0.2 to <0.6), moderate (ES of 0.6 to <1.2), large (ES of 1.2 to <2.0), very large (ES ≥ 2.0), and extremely large (ES > 4.0) [41]. Forest plots were utilized to illustrate point estimates of the effect size and 95% confidence intervals. A positive ES value in BDNF and a negative ES value in the psychomotor test (i.e., time to completion in seconds) indicated that (poly)phenols-rich supplementation enhanced outcomes. For the forest plots, each individual study is represented through a black square, and the overall effect is represented by a red diamond. Statistical heterogeneity was calculated by computing Q [42] and *I*^2^ [43]. When substantial heterogeneity was present (*I*^2^ values > 50%), a random-effect model was utilized over a fixed-effect model [43]. To identify potential sources of variance and of heterogeneity, moderator analysis was performed using subgroup analysis for categorical variables including the age group (older adults (55 years old and over) vs. young/middle aged group (18–55 years old)), intervention duration (acute vs. chronic), and human bioavailability of the used phenolic compounds (i.e., low (<9%) vs. medium (9–18%) vs. high (<18%)). Additionally, meta-regression for decimal variables (i.e., phenolic dose and percentage of female participants) and categorical variable (i.e., nature of phenolic compounds) was also performed. Funnel plots’ potential asymmetries, the Begg and Mazumdar’s rank correlation test [44], the Egger’s linear regression test [45], and the Duval and Tweedie’s trim-and-fill test [46] were used to examine publication bias. Sensitivity analyses and cumulative meta-analysis were also conducted to assess the stability and the reliability of the findings. Statistical significance was set at *p* < 0.05.

## 3. Results

### 3.1. Study Selection

The predefined search strategies yielded a preliminary pool of 4303 possible papers. A totalof 1688 duplicates and 2385 non-clinical trials were removed. Then, 230 published papers were screened by titles and abstracts for eligibility, and 38 published studies met the inclusion criteria. After a careful review of the 38 full texts, 15 articles included enough comparable data (based on the used cognitive test and the tested brain parameters) to be used in the present subgroup meta-analysis (Figure 1).

### 3.2. Study Characteristics

Fifteen studies [19,23,24,27,28,47,48,49,50,51,52,53,54,55,56] examining the effects of (poly)phenols-rich supplementation on psychomotor function and/or BDNF met the specific inclusion criteria and were included in the meta-analysis.

The characteristics of each study, as well as the effect of (poly)phenols-rich supplementation on psychomotor function and BDNF are summarized in Table 2 and Table 3, respectively. Nine papers [19,23,24,28,47,48,49,50,51] examined the effect of (poly)phenols-rich supplementation on psychomotor function (e.g., TMTa and RTT). Five studies [27,52,53,54,55,56] examined the change in serum BDNF following (poly)phenols-rich supplementation. Only one study examined the effect of (poly)phenol-rich supplementation on psychomotor functions, as well as on BDNF [27].

Regarding supplementation protocol, three studies used resveratrol [27,47,53], three studies used cocoa flavanols [19,28,51], and nine studies used one each of the following phenolic compounds: soy-extracted isoflavones [48], Ginkgo biloba extract [49], cocoa catechins [23], matcha tea powder [24], anthocyanin-rich purple grape juice [50], rich-anthocyanin blueberry [52], high-flavanol chocolate [54], flavonoid-rich Ginko biloba capsule [55], or green tea catechins [56].

Three studies used two different doses of (poly)phenols (high and low OR high and medium doses) [19,47,51]. Nine studies used only low doses of (poly)phenols [23,27,28,48,49,50,52,53,55]. Two studies used only high doses of (poly)phenols [24,54]. Only one study used a medium dose of (poly)phenols [56].

Phenolic compound with (i) low human bioavailability rates were used in seven studies [24,27,47,49,50,52,53,56], (ii) medium human bioavailability rates were used in seven studies [19,23,24,28,51,54,55], and (iii) high human bioavailability rates were used in one study [48].

Concerning the duration of the supplementation protocol, ten studies investigated the chronic effect (5 days to 26 weeks) [19,27,28,47,48,49,52,53,55,56], while four studies investigated the acute effect of (poly)phenols-rich supplementation on psychomotor functions and/or BDNF [24,50,51,54]. Only one study [23] investigated both acute and chronic effects of (poly)phenols-rich supplementation on psychomotor functions.

### 3.3. Subject Characteristics

A total of 522 participants were included in this systematic review and meta-analysis. The number of participants in each trial ranged from 12 to 60. Seven studies targeted a healthy older population with ages ranging from 60 to 93 years [19,27,47,48,49,52,53], while eight studies targeted a healthy young- and middle-age population with mean age ranging from 18 to 51 years [23,24,28,50,51,54,55,56]. In ten studies, participants from both genders were recruited [19,23,24,27,47,49,50,52,53]. Three studies have recruited only male participants [54,55,56], and two studies recruited only female participants [28,48].

### 3.4. Effect of (Poly)Phenols-Rich Supplementation on Psychomotor Functions and BDNF

Out of the 10 studies investigating the change in the psychomotor performances following acute and/or chronic supplementation of polyphenols-rich supplementation, four studies showed a significant improvement in psychomotor function (i.e., TMT or RRT completion time in s) compared to placebo (PLA) [19,24,47,50] (Table 2). Regarding the change in BDNF levels, only one study showed a significant improvement in BDNF levels following (poly)phenols-rich supplementation [55] (Table 3). 

### 3.5. Methodological Quality of Studies

Overall, the study quality was deemed to be high to excellent (Table S1). The Physiotherapy Evidence Database (PEDro) scale revealed a high score of eight and above for all included studies (mean ± SD = 8.9 ± 0.26), with 14 studies receiving a very high score of 9 out of 10 (i.e., a double-blind but not triple-blind trial). A score of 8 was given to one investigation as the authors failed to blind all assessors and to conceal allocation [47].

### 3.6. Meta-Analysis Results

#### 3.6.1. Effect of (Poly)Phenols-Rich Supplementation on Psychomotor Functions

**Figure 2:**
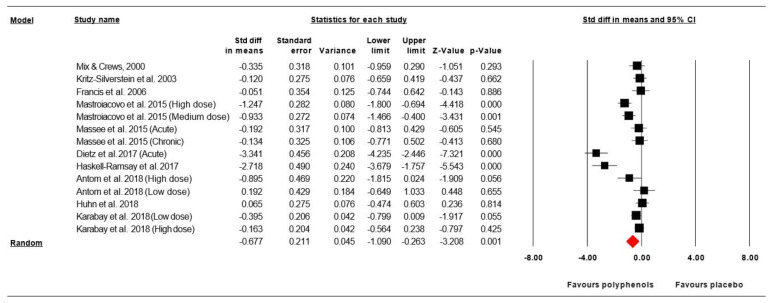
Forest plot of the standardized differences in means of the effect of (poly) phenols-rich supplementation on psychomotor functions. Note: A negative direction of effect size (ES) indicates a better effect of (poly)phenols-rich supplementation.

Data from ten studies investigating the effect of (poly)phenols-rich supplementation on psychomotor function were pooled in our MA. Since the studies of Antom et al. [47], Mastroicovo et al. [19], and Karabay et al. [51] included two phenolic doses and the study of Masse et al. [23] included two intervention duration, the results from each condition were considered as independent studies. The summarized effects of 14 ESs showed a moderate effect (ES = −0.677, SE = 0.211, 95% CI 1.090 to −0.263, *Z*-value = −3.208, *p* = 0.001; Figure 2) of (poly) phenols-rich supplementation on the psychomotor function. A significant heterogeneity was computed (Q = 85.248, df =13, *p* = 0.000; *I*^2^ = 84.750%). To identify potential sources of heterogeneity, a sub-analysis and meta-regression analysis were performed.

*Subgroup analysis:* A subgroup analysis for the categorical variable “participant age group” revealed that (poly)phenols-rich supplementation had a significant impact on psychomotor function in young/middle aged adults but not in older adults, with an SDM of −0.894 (Standard Error (SE) = 0.314, 95% CI −1.510 to 0.279, *Z*-value= −2.85, *p* = 0.004)) and -0.47 (SE = 0.313, 95% CI −1.083 to 0.143, *Z*-value = −1.504, *p* = 0.133), respectively (Figure S1).

A subgroup analysis for the categorical variable “intervention duration” revealed a significant impact on psychomotor function during acute (poly)phenols-rich supplementation (SDM = −1.023, SE = 0.339, 95% CI −1.688 to −0.359, *Z*-value = −3.018, *p* = 0.003), which was not the case using the chronic condition (SDM = −0.428, SE = 0.292, 95% CI −1.00 to 0.144, *Z*-value = −1.465, *p* = 0.143), respectively (Figure S2).

A subgroup analysis for the categorical variable “human bioavailability of phenolic compound” revealed a significant impact of (poly)phenols-rich supplementation on psychomotor function using phenolic compounds with medium human bioavailability (SDM = −0.760, SE = 0.297, 95% CI −1.341 to −0.178, *Z*-value = −2.562, *p* = 0.01); which was not the case using the low bioavailability compounds (SDM = −0.678, SE = 0.392, 95% CI −1.446 to 0.091, *Z*-value = −1.728, *p* = 0.084), respectively (Figure S3).

**Figure 3:**
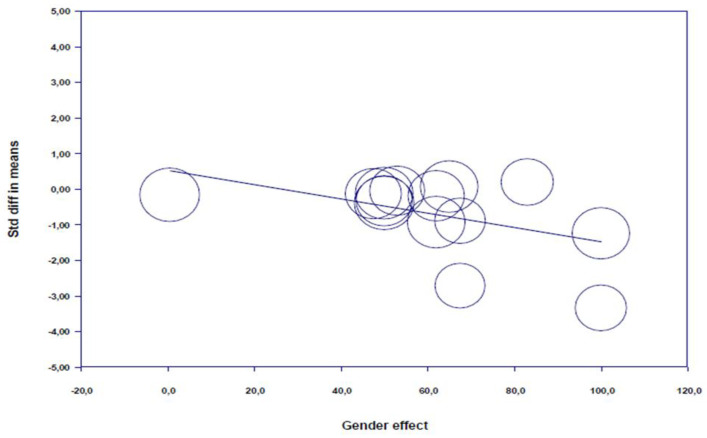
Scatter plot of regression analysis showing the influence of polyphenols supplementation by gender.

*Meta-regression:* The regression analysis showed only participant gender (*p* = 0.013, Figure 3, Table S1) was a significant predictor of (poly)phenols-rich supplementation effects on psychomotor functions. However, after (poly)phenols-rich supplementation, psychomotor performances are not able to be predicted from the phenolic dose (*p* = 0.453), and the “phenolic compound nature” (*p* = 0.267, Table S2).

These data suggest (poly)phenol-rich supplementation has a greater positive impact on psychomotor functions (i) in younger individuals, (ii) after acute usage, (iii) when utilizing compounds with rates of medium bioavailability, and (vi) when studies included a greater percentages of females.

**Figure 4:**
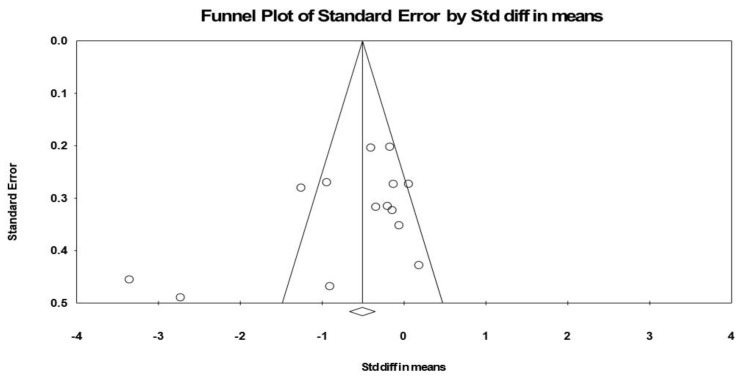
Funnel plot of psychomotor performance following (poly)phenols-rich supplementation, showing evidence of publication bias.

*Publication bias:* Visual inspection of the funnel plot (Figure 4) and the performance of the Egger’s linear regression test (intercept = −4.519, SE = 2.385, 95% CI −9.715 to 0.677, *t* = 1.895, df = 12, *p* = 0.041) showed evidence of publication bias. However, the Begg and Mazumdar’s rank correlation test (Kendall’s S statistic *p* − Q = −25.00; tau without continuity correctio*n* = −0.275, z = 1.369, *p* = 0.086; tau with continuity correctio*n* = −0.264, z = 1.314, *p* = 0.094) showed the lack of publication bias. With the Duval and Tweedie trim-and-fill analysis, three studies [27,29,48] were trimmed, resulting in a “true ES” of −0.885.

*Sensitivity and cumulative meta-analysis*: Both sensitivity analysis and cumulative meta-analysis confirmed the reliability and stability of the current findings (Figure S4).

#### 3.6.2. Effect of (Poly)Phenols-Rich Supplementation on BDNF

**Figure 5:**
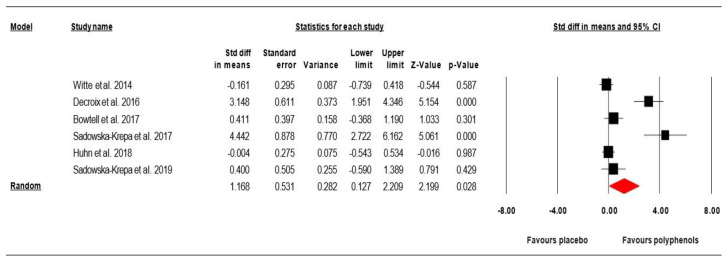
Forest plot including standardized differences in means of effects of (poly)phenols-rich supplementation on BDNF concentrations. Note: Positive direction of ES is indicative a greater effects from (poly)phenols-rich supplementation.

Data from six studies investigating the effect of (poly)phenols-rich supplementation on BDNF were pooled in our MA. The summarized effects of six ESs showed a moderate effect (ES = 1.168, SE = 0.531, 95% CI 0.127 to 2.209, *Z*-value = 2.199, *p* = 0.028; Figure 5) of (poly)phenols-rich supplementation on the BDNF. A significant heterogeneity was computed (Q = 47.199, df = 5, *p* = 0.000; *I^2^* = 89.407%); therefore, a sub-analysis and meta-regression analysis were performed.

*Subgroup analysis:* A subgroup analysis for the categorical variable “participant age group” revealed that (poly)phenols-rich supplementation had a significant impact on BDNF in young/middle aged adults, but not in older adults, with an SDM of 2.409 (SE = 0.629, 95% CI 1.176 to 3.641, *Z*-value = 3.831, *p* = 0.000) and 0.073 (SE = 0.536, 95% CI −0.977 to 1.123, *Z*-value = 0.136, *p* = 0.892), respectively (Figure S5).

A subgroup analysis for the categorical variable “human bioavailability of phenolic compound” revealed a significant impact of (poly)phenols-rich supplementation on BDNF using phenolic compounds with medium human bioavailability (SDM = 3.570, SE = 0.501, 95% CI 2.588 to 4.553, *Z*-value = 7.121, *p* = 0.000), which was not the case using the low bioavailability compounds (SDM = 0.065, SE = 0.169, 95% CI −0.267 to 0.396, *Z*-value = 0.383, *p* = 0.701), respectively (Figure S6).

Regarding the categorial variable “intervention duration”, it was not possible to do the subgroup analysis given that only one study [54] employed acute intervention strategy.

**Figure 6:**
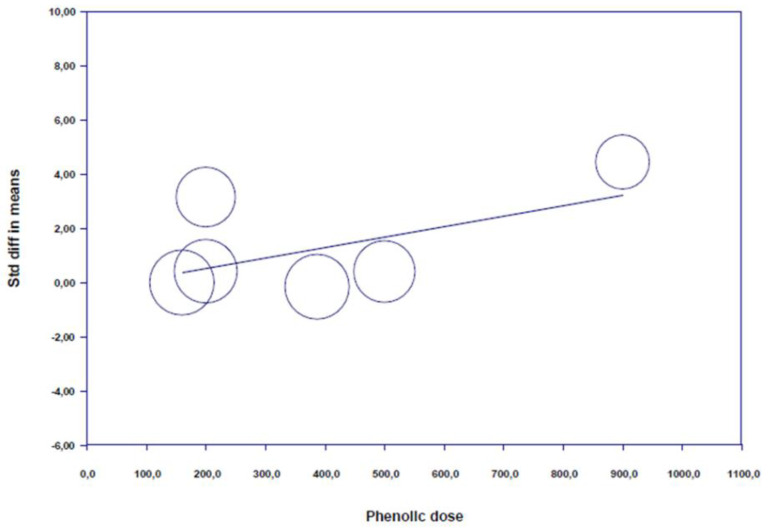
Scatter plot of regression analysis showing the influence of polyphenols supplementation by phenolic dose.

*Meta-regressions:* A regression analysis demonstrated “phenolic dose” (*p* = 0.093, Figure 6, Table S2), and “gender” (*p* = 0.982, Table S3) is not able to predict BDNF concentrations after (poly)phenol-rich supplementation. However, a scatter plot of phenolic dose related regression results (Figure 6) was skewed toward utilizing a medium/high dose for higher beneficial impacts.

Regarding the categorical variable “nature of phenolic compound”, it was not possible to do the meta-regression, given the very low number of categories: only five phenolic compounds (i.e., resveratrol, anthocyanin-rich blueberry, high-flavanol chocolate, flavonoid-rich Ginko biloba capsule, green tea catechins).

These data suggest that (poly)phenol-rich supplementation has greater efficacious impacts on brain plasticity (i) in younger individuals, (ii) using phenolic compounds with at least medium rates of bioavailability, and (iii) using greater doses of polyphenols.

**Figure 7:**
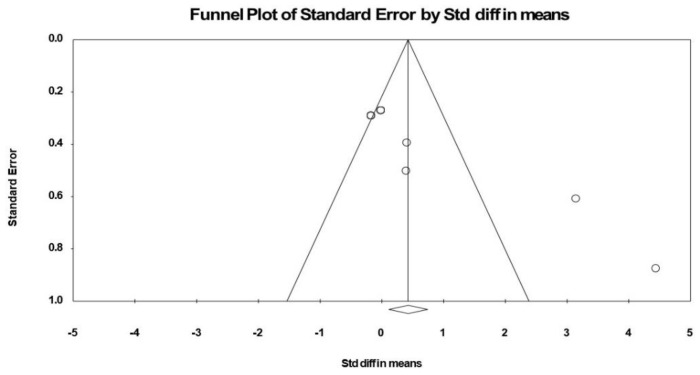
Funnel plot of BDNF following (poly)phenols-rich supplementation, showing evidence of publication bias.

*Publication bias:* Visual inspection of the funnel plot (Figure 7), the performance of the Egger’s linear regression test (intercept = 7.243, SE = 1.618, 95% CI 2.750 to 11.736, *t* = 4.475, df = 4, *p* = 0.005), and the Begg and Mazumdar’s rank correlation test (Kendall’s S statistic *p* − Q = 11.00, tau without continuity correctio*n* = 0.733, z = 2.066, *p* = 0.019, tau with continuity correctio*n* = 0.666, z = 1.879, *p* = 0.03) showed evidence of publication bias. However, the Duval and Tweedie’s trim-and-fill test did not identify any missing study.

*Sensitivity and cumulative meta-analysis:* Stability and reliability of current findings are confirmed via sensitivity analysis and cumulative meta-analysis (Figure S7).

## 4. Discussion

Recent research in nutritional neuroscience has underlined the importance of (poly)phenols-rich supplementation for the maintenance of physiological balance [57,58] and thereby possible beneficial effects on human brain and cognitive functions [13,15,16,59]. The purpose of the present work was to conduct a systematic review and meta-analysis of studies evaluating the effects of (poly)phenols-rich supplementation on cognitive functions and brain parameters in humans and to examine possible moderator variables (i.e., related to the participants characteristics and the supplementation protocol) of this relationship. Two main items (i.e., psychomotor function and BDNF) were sufficiently comparable and included in the MA.

The reviewed studies support the beneficial effect of (poly)phenols-rich supplementation on (i) psychomotor functions with faster completion time in RTT and TMTa, and (ii) brain plasticity biomarkers with higher BDNF level using (poly)phenols-rich supplementation compared to placebo. Main moderator variables of these beneficial effects seem to be participant age, gender, used phenolic compounds, and human bioavailability rate.

### 4.1. Effect of (Poly)Phenols-Rich Supplementation On Psychomotor Functions and Moderator Variables

The pooled analysis suggests that (poly)phenols-rich supplementation has a significant effect on psychomotor functions. Particularly, the administration of medium (520 mg) to high (993 mg) doses of cocoa flavanols for 8 weeks did speed up the completion of the TMTa test in older adults [19]. Similarly, a significant reduction in completion time during the RTT (i.e., faster reaction time) was revealed in young/middle-aged adults following the acute administration of low doses of EGCG matcha tea [24] or of an anthocyanin-PGj [50].

However, no significant effects were observed for the (i) chronic administration (6–26 weeks) of low resveratrol [27,47], soy-extracted isoflavones [48], or Ginkgo biloba extract EGb [49] doses in older adults and (ii) acute administration of low catechin cocoa extract [23], and low or high cocoa flavanols [51], in young/middle aged adults. Discrepancies between findings were previously related to the adopted supplementation protocol and to participant characteristics [15,16].

A subgroup analysis of participant age, the intervention duration, and the human bioavailability of the used (poly)phenols revealed differences in psychomotor performance. (Poly)phenol-rich supplementation had a higher beneficial impact (i) in the younger population compared to the older one (SDM = −0.89 vs. −0.47), (ii) following an acute compared to chronic supplementation (SDM = −1.02 vs. −0.43), and (iii) using a phenolic compound with medium compared to low bioavailability rates (SDM = −0.76 vs. −0.68). The results of the intervention duration could be explained by the age group of the recruited participants, as the majority of participants in the acute intervention were young- or middle-aged adults, while those recruited in the chronic intervention were older adults. Therefore, further studies should specifically investigate acute and chronic protocols in homogeneous populations from an age prospective.

The meta-analysis showed also a high heterogeneity between the results of the included studies (*I*^2^ = 84.75%). Meta-regressions demonstrated statistically significant moderation effects of participant gender as studies including higher percentages of female participants demonstrated greater improvement in psychomotor functions. However, no significant moderation effect was shown for the phenolic compounds and doses.

The subgroup analysis and the meta-regression demonstrated the impact of the trial methodology indicating that younger adults respond better to (poly)phenols intervention and that (poly)phenolic compounds with a medium human bioavailability rate may be more advantageous in terms of improving psychomotor performance. These findings could (at least partially) explain the contradictory findings in previous studies [15,16] and the high heterogeneity reported in this meta-analysis.

The beneficial effects of (poly)phenols on cognitive performance may be due to their positive impact on limiting nitric oxide (NO) scavenging by ROS, thereby enhancing NO bioavailability and activating NO synthesis (NOS) pathways [15,16,60], which are important contributors to flow-mediated dilation [61,62] and neurotransmission [63]. As NOS is responsible for vasodilation [64], promoting NOS via (poly)phenols-rich supplementation will increase regional perfusion, brain activity, and may contribute to improved cognitive performance [15,16,51]. Similarly, as NO acts as a neurotransmitter [65], enhanced cognition following (poly)phenols consumption has been also explained by the improved neuronal signaling pathways via activated NOS [16,51,63].

### 4.2. Effect of (Poly)Phenols-Rich Supplementation on BDNF and Moderator Variables

It is well documented that brain plasticity plays an important role in cognitive function [66] with increased BDNF levels in the brain seeming to stimulate synaptic plasticity and neurogenesis, thus suggesting the ability to enhance cognition [66,67]. The pooled findings of the present MA support this hypothesis and show that the significant beneficial effect of (poly)phenols-rich supplementation on psychomotor functions was accompanied with improved BDNF levels.

The acute administration of a high dose of flavanol-rich chocolate [54], or the chronic administration (6 weeks) of a low dose of flavonoid-rich Ginkgo biloba [55], led to enhanced BDNF levels in young/middle aged adults. However, no significant effects were observed for the chronic administration (6–26 weeks) of a low dose of resveratrol [27,53], or anthocyanin-rich blueberry [52], in older adults as well as following the chronic administration of a medium-rich catechin green tea dose in young/middle aged adults [56]. The discrepancies between findings were confirmed by a high heterogeneity observed between the results of the included studies (*I*^2^ = 89.41%).

In accordance with the findings of the psychomotor performance, the subgroup analysis of the participants’ age group and the (poly)phenols bioavailability in the BDNF-related study revealed differences in BDNF levels with a higher beneficial impact of (poly)phenols-rich supplementation in the younger population compared to the older (SDM = 2.41 vs. 0.07) as well as using phenolic compounds with medium compared to low bioavailability rate (SDM = 3.57 vs. 0.07). There are no available results for the effects of intervention duration, as only one study employed an acute intervention.

Meta-regressions revealed non-significant moderation effects of phenolic dose and participants gender. However, a skewed scatter plot toward a greater impact using higher doses was observed.

The subgroup analysis and the meta-regression demonstrated, again, the impact of the trial methodology and indicated that in terms of enhancing BDNF levels, (i) younger and male participants respond better to (poly)phenol interventions, and (ii) (poly)phenolic compounds with a medium human bioavailability rate showed greater impact. These findings could help better understanding recent suggestions that contradictory findings in nutritional neuroscience could be related to supplementation protocols and participant characteristics [15,16].

The exact mechanism behind the beneficial effect of (poly)phenols-rich supplementation in relation to brain plasticity is yet to be determined [13]. However, a number of potential mechanisms, such as the activation of the NADPH oxidase pathway [68] and the induced synaptic plasticity [69] via modulation of receptor function, gene expression, and interaction with signaling pathways [70], have been proposed to explain the aforementioned beneficial impact of (poly)phenol on BDNF levels.

Taken together, the subgroup analysis and the meta-regression in both psychomotor and BDNF-related studies indicate that younger males seem to be the best responders to (poly)phenol supplementation. These findings support previous data that (poly)phenol interventions for brain-related aging processes are more advantageous at a younger age [16]. Additionally, it confirms that young people may be the most attractive population for interventions targeting health span extension [30]. As organs are less damaged in the young- and middle-aged population compared to older adults, it is theoretically easier to improve cognitive functions and brain plasticity, thereby reducing the onset of brain related aging-process by applying early anti-aging interventions [31].

Regarding gender differences, it is well documented that gender has a moderating effect on cognition and the brain-aging process [33]. The present subgroup analysis confirms this moderating effect during antiaging (poly)phenol interventions, demonstrating that men may benefit more than women as it pertains to the beneficial impact of (poly)phenol supplementation on cognitive functions.

In terms of supplementation protocol, it seems that an optimal anti-aging (poly)phenol intervention should include a phenolic compound with at least a medium human bioavailability rate. The present subgroup analysis of medium bioavailability rates revealed significant impacts on psychomotor and BDNF results, while those with low bioavailability rates demonstrated non-significant impacts. Subgroup analyses for high bioavailability rates were not possible given the lack of available literature. Previous reports indicate that a sufficient amount of (poly)phenol metabolites should cross the blood–brain barrier toward their specific binding sites on neurons to exert beneficial effects on the brain [15,16,71,72]. As the human bioavailability rate reflects the extent to which the bioactive compound is absorbed and becomes available at the site of action in an appropriate amount of time [73], the bioavailability rate of the consumed phenolic compound (ranging from 0.3% to 43% [32]) has been previously suggested as an important factor influencing the impact on and change in cognition and brain functions [15,16,59,72]. Findings of the present subgroup analysis confirm this hypothesis and show that a low bioavailability of phenolic compounds masks the beneficial impact of (poly)phenols-rich supplementation.

Regarding optimal dosage, although a non-significant effect was found for this variable, the skewness of the meta-regression results toward a medium/high dose for higher beneficial impacts suggest that a medium to high dose (≈500–1000 mg) of (poly)phenols may result in more pronounced positive effects on brain plasticity. However, caution must be taken when interpreting these results due to pharmacological differences between (poly)phenols. While the dose-related sub-analysis incorporated all (poly)phenolic compounds, individual pharmacokinetic and pharmacodynamic differences subsist [15,16]. Additionally, given that only one study investigated the effect of high (poly)phenols dose (1000 mg) on BDNF [47], more studies comparing the impact of moderate, high, and very high doses are needed to identify the exact optimal dose. Indeed, a previous report indicate that compared to medium dosing, high dosing of (poly)phenols can result in decreased fractional absorption, inducing saturable mechanism and limiting glucosides uptake [74]. Therefore, future studies should identify, specifically for glycosylated polyphenols, the optimal dose that enhances brain plasticity without being compromised by saturation processes.

## 5. Strengths and Weaknesses

The strengths of this systematic review and meta-analysis are (i) the inclusion of different age groups and gender, as well as differing supplementation protocols (i.e., durations using different dose of phenolic compounds with low, medium, or high human bioavailability), (ii) the assessment of the moderation effects of these different variables using subgroup analysis or meta-regression, and (iii) the comprehensive coverage of the literature followed by careful appraisal of the included studies’ quality. Interpretation of the results of the meta-analysis involving studies in psychomotor and BDNF responses is challenged by the significant amount of heterogeneity and the evidence of publication bias in the selected psychomotor papers. Weaknesses of this study are that (i) the majority of psychomotor studies in older adults have used chronic intervention, while the majority of studies in younger adults adopted acute supplementation protocols, (ii) only one study evaluated the effect of phenolic compounds with high bioavailability rates on psychomotor function, and (iii) only one study evaluated the acute effect of (poly)phenols-rich supplementation in BDNF. Therefore, all results must be interpreted with caution, due to the lack of studies showing comparable results.

## 6. Conclusions

The present systematic review and meta-analysis support the beneficial effect of (poly)phenol-rich foods on humans’ psychomotor function and brain plasticity in healthy adults, showing a significant impact of this nutritional anti-aging strategy in improving RTT or TMTa performances, as well as in enhancing BDNF level compared to placebo. Subgroup analysis and meta-regression indicated that these beneficial effects appear to depend on the target population (i.e., age group and gender) and the adopted supplementation protocol (i.e., phenolic compounds and its bioavailability), with a significant beneficial effect observed in studies targeting young/middle-aged adults, female participants, and phenolic compounds with a medium bioavailability rate. It also appears that more beneficial effects (non-significant skewed scatter plot) can be observed using a medium to high (poly)phenol dose. In conclusion, the present results suggest age group and gender, the used phenolic compounds and its human bioavailability rate, and the supplementation dose are the primary moderator variables relating to the beneficial effects of (poly)phenol consumption on humans’ cognitive and brain function. Therefore, it seems more advantageous to begin anti-aging (poly)phenol interventions in adults at a younger age using medium to high dose of phenolic compounds, with at least medium bioavailability rate. More beneficial effects can be expected in female participants who showed higher responsiveness to (poly)phenol intervention. These findings provide better insight into (poly)phenols’ impact on psychomotor functions and brain plasticity, and they provide clear guidelines to design an optimized protocol for future anti-aging interventions. However, as the number of available studies concerning the described topic was rather small, more rigorous research comparing the effect of different bioavailability (poly)phenol compounds at different doses are needed in both younger and older adults, as well as both men and women, to confirm these results.

## Figures and Tables

**Table 1:** A summary of the search strategy and the inclusion and exclusion criteria adopted in the present systematic review and meta-analysis.

Search Strategy Item	Search Strategy Details
String of keywords	((polyphenol) OR (flavonoids) OR (polyphenolic compounds) OR (isoflavone) OR (flavanol) OR (phytoestrogen) OR (resveratrol)) AND ((cognitive performance) OR (cognitive) OR (cognitive function) OR (cognition) OR (brain function) OR (executive function) OR (attention) OR (working memory) OR (brain imaging) OR (neuroimaging) OR (neural) OR (magnetic resonance imaging) OR (MRI) OR (fmri) OR (grey matter) OR (gray matter) OR (brain volume) OR (brain structure) OR (electrophysiology) OR (EEG) OR (event related potential) OR (neuroblast) OR (neuroblast) OR (cerebral blood flow) OR (CBF) OR (regional perfusion) OR (brain-derived neurotrophic factor) OR (BDNF) OR (cerebrovascular responsiveness) OR (CVR) OR (pulsatility index) OR (transcranial doppler) OR (TCD) OR (near-infrared spectroscopy) OR (NIRS) OR (cerebral hemodynamics) OR (total hemoglobin) OR (total-Hb) OR (oxygenated hemoglobin) OR (oxy-Hb) OR (deoxygenated 2 hemoglobin) OR (deoxy-Hb)) NOT ((mice) OR (animals) OR (Parkinson’s) OR (stroke) OR (Alzheimer’s) OR (dementia) OR (cancer) OR (lesions) OR (diabetes) OR (injury) OR (patients) OR (rats) OR (disease) OR (impairment)]
Searched databases	Web of Science and PubMed; up to July 2019
Inclusion criteria	(i) English language published primary research (up to July 2019), (ii) research in healthy adult humans, (iii) original investigations researching effects of (poly)phenol-rich supplementation on brain health, (iv) no major methodological issues (i.e., lack of a comparative control, not blinded, or inappropriate/ incorrect statistical analyses)
Exclusion criteria	(i) studies written in any non-English language, (ii) congress, meeting, conference, or workshop publications, (iii) studies conducted in diseased individuals or a individuals greater than 55 years of age and (iv) studies that did not include supplementation. Findings from sources such as encyclopedias, reviews, case studies, or book chapters were not included.
Time filter	None applied (search from inception)
Language filter	English
PICOS	Participants: healthy adults (>18 years of age) Intervention: chronic and/or acute (poly)phenols-rich supplementation Comparative: Any Outcome: cognitive function (e.g., neuroplasticity, overall cognition, executive function, processing speed, verbal memory, language psychomotor performance, visual memory, attention) and brain activity, neuroprotective measures (e.g., brain perfusion, cerebral blood flow (CBF), cerebral hemodynamics, and neuroinflammation) Study design: controlled clinical trial

**Table 2:** Effects of (poly)phenols-rich supplementation on psychomotor functions.

Age Group	Authors	Study Design	Participants Characteristics			Supplementation Protocol				Effect on Psychomotor Functions
			Number of Participants	Age of Participants	Gender (% of Female)	Phenolic Compounds	Dose	Bioavailability	Intervention Duration	
Old-aged Adults	Antom et al. [47]	Double-blind, randomized PLA-controlled trial	*n* = 32 (10 PLA, 12 low dose, 10 high dose)	Mean age: 73.34 ± 7.02 years old (65–93 years)	50%	Resveratrol	High dose: 1000 mg/day	Low	Chronic: 12 weeks	Psychomotor speed improved on the TMT (a) compared to PLA (TMT in s)
							Low dose:300 mg/day			Non-significant effect on psychomotor speed on the TMT (a) compared to PLA (TMT in s)
	Huhn et al. [27]	Double-blind, randomized PLA-controlled trial	*n* = 60 (30 resveratrol group, 30 PLA)	Age: 60–79 years	53%	Resveratrol	Low dose: 200 mg/day	Low	Chronic: 26 weeks	Non-significant effect on psychomotor speed on the TMT (a) compared to PLA (TMT in s)
	Kritz-Silverstein et al. [48]	Double-blind, randomized PLA-controlled trial	*n* = 53 (27 treatment, 26 PLA)	Mean age: SOY-ISF = 60 ± 4, PLA= 62 ± 6 years	100%	Soy-extracted isoflavones	Low dose: 110 mg/day	High: 43%	Chronic: 26 weeks	Non-significant effect on psychomotor speed on the TMT (a) compared to PLA (TMT in s)
	Mastroiacovo et al. [19]	Double-blind, controlled, parallel-arm trial	*n* = 90 (30 for each study’s arm: high, moderate, low, flavanol)	Age >60 years old	62%	Cocoa flavanols	High dose: 993 mg	Medium	Chronic: 8 weeks	Psychomotor speed improved on the TMT (a) compared to PLA (TMT in s)
							Medium dose: 520 mg			Psychomotor speed improved on the TMT (a) compared to PLA (TMT in s)
	Mix & Crews. [49]	Double-blind, PLA-controlled, parallel-group trial	*n* = 48 (n of each arm: not mentioned)	Age range: 55–86 years old	47.50%	Ginkgo biloba extract EGb 761	Low dose: 180 mg/day	Low	Chronic: 6 weeks	Non-significant effect on psychomotor speed on the TMT (a) compared to PLA (TMT in s)
Young- and middle-aged adults	Francis et al. [28]	A double blind counterbalanced	*n* = 16	Age range: 18–30 years old	100%	Cocoa flavanols	Low dose: 172 mg/day	Medium	5 days	Non-significant effect on psychomotor speed on the RTT compared to PLA (RTT in s)
	Massee et al. [23]	Randomized, PLA-controlled, double-blind, parallel design	*n* = 38	Mean age: 24.13 ± 4.47 years old (18–40 years)	67.50%	Catechin cocoa extract	Low dose 250 mg/day	Medium: 18%	Acute	Non-significant effect on psychomotor speed on the RTT compared to PLA (RTT in s)
									Chronic: 4weeks	Non-significant effect on psychomotor speed on the RTT compared to PLA (RTT in s)
	Dietz et al. [24]	Randomized, single-blind, PLA-controlled, counterbalanced trial	*n* = 23	Mean age: 24.7 years old (20–35 years)	83%	Matcha tea powder	High dose: 4 g/day	Low	Acute	Psychomotor speed improved on the RTT compared to PLA (RTT in s)
	Haskell-Ramsay et al. [50]	Randomized, PLA-controlled, double-blind, counterbalanced-design	*n* = 20	Mean age: 21.1 years old	65%	Anthocyanin-rich purple grape juice	Low dose: 138 mg/day	Low	Acute	Psychomotor speed improved on the RTT compared to PLA (RTT in s)
	Karabay et al. [51]	Randomized, double-blind, PLA-controlled counterbalanced design	*n* = 24	Mean age: 22.2 years old (18–29 years)	50%	Cocoa flavanols	Low dose: 374 mg/day	Medium	Acute	Non-significant effect on psychomotor speed on the RTT compared to PLA (RTT in s)
							High dose: 747 mg/day			Non-significant effect on psychomotor speed on the RTT compared to PLA (RTT in s)

Placebo (PLA), Trail Making Test (TMT), Reaction Time Test (RTT), soy-extracted isoflavones (SOY-ISF).

**Table 3:** Effects of (poly)phenols-rich supplementation on brain-derived neurotrophic factor.

Age Group	Author	Study Design	Participants Characteristics			Supplementation Protocol				Effect on BDNF
			Number of Participants	Age of Participant	Gender (% of Female)	Phenolic Compound	Dose	Bioavailability	Intervention Duration	
Old-aged Adults	Bowtell et al. [52]	Randomized, double-blind, PLA-controlled parallel trial	*n* = 26 (12 blueberry, 14 PLA)	Mean age: BB group = 67.5 ± 0.9, PLA group = 69 ± 0.9	42%	Anthocyanin-rich blueberry	Low dose: 387 mg/day	Low	Chronic: 12 weeks	Non-significant effect on BDNF compared to PLA (BDNF in (ng/mL))
	Huhn et al. [27]	Double-blind, randomized PLA-controlled trial	*n* = 60 (30 resveratrol group, 30 PLA)	Age: 60–79 years	53%	Resveratrol	Low dose: 200 mg/day	Low	Chronic: 26 weeks	Non-significant effect on BDNF compared to PLA (BDNF in (ng/mL))
	Witte et al. [53]	Double blind, randomized, PLA-controlled, parallel groups study	*n* = 46 (23 resveratrol, 23 PLA)	Mean age: RESV group = 65 ± 7, PLA = 64 ± 5 years old	39%	Resveratrol	Low dose: 200 mg/day	Low	Chronic: 26 weeks	Non-significant effect on BDNF compared to PLA (BDNF in (ng/mL))
Young- and middle-aged adults	Decroix et al. [54]	Randomized, double-blind, PLA-controlled, counterbalanced design	*n* = 12	Mean age: 30 ± 3 years old	0%	Flavanol-rich chocolate	High dose: 900 mg/day	Medium	Acute	Non-significant effect on BDNF compared to PLA (BDNF in (ng/mL))
	Sadowska-Krępa et al. [55]	Randomized, double-blind, placebo-controlled, parallel-groups study	*n* = 18	Age range: 18–25 years old	0%	Flavonoid-rich Ginko biloba capsule	Low dose: 160 mg/day	Medium	Chronic: 6 weeks	BDNF significantly improved compared to PLA (BDNF in (ng/ml))
	Sadowska-Krępa et al. [56]	Randomized, double-blind, placebo-controlled, parallel-groups study	*n* = 16	Age range: 18–25 years old	0%	Catechin-rich green tee	Medium dose: 500 mg/day	Low	Chronic: 6 weeks	Non-significant effect on BDNF compared to PLA (BDNF in (ng/ml))

Placebo (PLA), blueberry (BB), Brain-derived neurotrophic factor (BDNF), Resveratrol (RESV).

## Supplementary Materials

The following are available online at https://www.mdpi.com/2072-6643/12/9/2872/s1, **Table S1.** Methodological quality of the studies with (poly)phenols-rich supplementation assessed with the PEDro scale. **Table S2.** Meta-regression for gender, phenolic dose and nature of phenolic compound to predict the effect of (poly) phenols-rich supplementation on psychomotor functions. **Table S3.** Meta-regression for gender, phenolic dose and nature of phenolic compound to predict the effect of (poly) phenols-rich supplementation on BDNF blood concentrations. **Figure S1.** Plot of studies investigating the effect of (poly)phenols-rich supplementation on psychomotor function in old- and young/middle-aged adults. **Figure S2.** Plot of studies investigating the acute and chronic effect of (poly) phenols-rich supplementation on psychomotor function. **Figure S3.** Plot of studies investigating the effect of (poly) phenols-rich supplementation with low and medium human bioavailability on psychomotor function. **Figure S4.** Cumulative statistics and statistics with study removed for studies investigating the effect of (poly)phenols-rich supplementation on psychomotor functions. **Figure S5.** Plot of studies investigating the effect of (poly) phenols-rich supplementation on BDNF in old- and young/middle-aged adults. **Figure S6.** Plot of studies investigating the effect of (poly)phenols-rich supplementation with low and medium human bioavailability on BDNF. **Figure S7.** Cumulative statistics and statistics with study removed for studies investigating the effect of (poly)phenols-rich supplementation on BDNF.
