# Supplementary material for: Moderators of the Impact of (Poly)Phenols Interventions on Psychomotor Functions and BDNF: Insights from Subgroup Analysis and Meta-Regression
Source: Nutrients. 2020 Sep 19;12(9):2872. doi: 10.3390/nu12092872 (PMC7551086; doi:10.3390/nu12092872)
Supplement: Supplementary file 1 [file nutrients-12-02872-s001.pdf]

**Table S1.** Methodological quality of the studies with (poly)phenols-rich supplementation assessed with the PEDro scale.

| | Items | Antom et al. [48] | Huhn et al. [27] | Kritz-Silverstein et al. [49] | Mastroiacovo et al. [19] | Mix & Crews. [50] | Bowtell et al. [54] | Witte et al. [55] | Francis et al. [28] | Massee et al. [23] | Dietz et al. [51] | Haskell Ramsay et al. [52] | Karabay et al. [53] | Decroix et al. [56] | Sadowska-Krępa et al. [57] | Sadowska-Krępa et al. [58] |
|---|---|---|---|---|---|---|---|---|---|---|---|---|---|---|---|---|
| 1 | Eligibility criteria were specified | + | + | + | + | + | + | + | + | + | + | + | + | + | + | + |
| 2 | Subjects were randomly allocated to groups (in a crossover study, subjects were randomly allocated an order in which treatments were received) | + | + | + | + | + | + | + | + | + | + | + | + | + | + | + |
| 3 | Allocation was concealed | - | + | + | + | + | + | + | + | - | + | + | + | + | + | + |
| 4 | The groups were similar at baseline regarding the most important prognostic indicators | + | + | + | + | + | + | + | + | + | + | + | + | + | + | + |
| 5 | There was blinding of all subjects | + | + | + | + | + | + | + | + | + | + | + | + | + | + | + |
| 6 | There was blinding of all therapists who administered the therapy | + | + | + | + | + | + | + | + | - | + | + | + | + | + | + |
| 7 | There was blinding of all assessors who measured at least one key outcome | - | - | - | - | - | - | - | - | - | - | - | - | - | - | - |

| | | | | | | | | | | | | | | | | |
|---|---|---|---|---|---|---|---|---|---|---|---|---|---|---|---|---|
| 8 | Measures of at least one key outcome were obtained from more than 85% of the subjects initially allocated to groups | + | + | + | + | + | + | + | + | + | + | + | + | + | + | + |
| 9 | All subjects for whom outcome measures were available received the treatment or control condition as allocated or, where this was not the case, data for at least one key outcome was analysed by "intention to treat" | + | + | + | + | + | + | + | + | + | + | + | + | + | + | + |
| 10 | The results of between-group statistical comparisons are reported for at least one key outcome | + | + | + | + | + | + | + | + | + | + | + | + | + | + | + |
| 11 | The study provides both point measures and measures of variability for at least one key outcome | + | + | + | + | + | + | + | + | + | + | + | + | + | + | + |
| | Total score | 8 | 9 | 9 | 9 | 9 | 9 | 9 | 9 | 7 | 9 | 9 | 9 | 9 | 9 | 9 |

**Table S2.** Meta-regression for gender, phenolic dose and nature of phenolic compound to predict the effect of (poly) phenols-rich supplementation on psychomotor functions.

| Variables | Psychomotor functions | | | | | |
|---|---|---|---|---|---|---|
| **Decimal** | **Coefficient** | **SE** | **95% lower CI** | **95% upper CI** | **Z-value** | **P value** |
| Gender | -0.02 | 0.008 | -0.036 | 0.004 | -2.48 | 0.013 |
| Phenolic dose | 0.001 | 0.001 | -0.001 | 0.002 | -0.75 | 0.453 |
| **Categorical** | **Q** | | **df** | | **P value** | |
| Nature of phenolic compound | 7.62 | | 6 | | 0.267 | |

Abbreviations: SE: standard error, CI: confidence interval.

**Table S3.** Meta-regression for gender, phenolic dose and nature of phenolic compound to predict the effect of (poly) phenols-rich supplementation on BDNF blood concentrations.

| Variables | BDNF | | | | | |
|---|---|---|---|---|---|---|
| **Decimal** | **Coefficient** | **SE** | **95% lower CI** | **95% upper CI** | **Z-value** | **P value** |
| Gender | 0.001 | 0.028 | –0.054 | 0.055 | 0.02 | 0.982 |
| Phenolic dose | 0.004 | 0.002 | –0.001 | 0.008 | 1.68 | 0.093 |
| **Categorical** | **Q** | | **df** | | **P value** | |
| Nature of phenolic compound | | | NA | | | |

Abbreviations: NA: not applicable, SE: standard error, BDNF: Brain-Derived Neurotrophic Factor, CI: confidence interval.

| Group by subgroup within study | Study name | Participant's age group | Std diff in means | Standard error | Variance | Lower limit | Upper limit | Z-Value | p-Value |
|---|---|---|---|---|---|---|---|---|---|
| Older | Mix & Crews, 2000 | Older | -0.335 | 0.318 | 0.101 | -0.959 | 0.290 | -1.051 | 0.293 |
| Older | Kritz-Silverstein et al. 2003 | Older | -0.120 | 0.275 | 0.076 | -0.659 | 0.419 | -0.437 | 0.662 |
| Older | Mastroiacovo et al. 2015 (High dose) | Older | -1.247 | 0.282 | 0.080 | -1.800 | -0.694 | -4.418 | 0.000 |
| Older | Mastroiacovo et al. 2015(Medium dose) | Older | -0.933 | 0.272 | 0.074 | -1.466 | -0.400 | -3.431 | 0.001 |
| Older | Antomet al. 2018 (High dose) | Older | -0.895 | 0.469 | 0.220 | -1.815 | 0.024 | -1.909 | 0.056 |
| Older | Antomet al. 2018(Low dose) | Older | 0.192 | 0.429 | 0.184 | -0.649 | 1.033 | 0.448 | 0.655 |
| Older | Huhn et al. 2018 | Older | 0.065 | 0.275 | 0.076 | -0.474 | 0.603 | 0.236 | 0.814 |
| Older | | | -0.470 | 0.313 | 0.098 | -1.083 | 0.143 | -1.504 | 0.133 |
| Young-middle | Francis et al. 2006 | Young-middle | -0.051 | 0.354 | 0.125 | -0.744 | 0.642 | -0.143 | 0.886 |
| Young-middle | Massee et al. 2015(Acute) | Young-middle | -0.192 | 0.317 | 0.100 | -0.813 | 0.429 | -0.605 | 0.545 |
| Young-middle | Massee et al. 2015(Chronic) | Young-middle | -0.134 | 0.325 | 0.106 | -0.771 | 0.502 | -0.413 | 0.680 |
| Young-middle | Dietz et al. 2017 | Young-middle | -3.341 | 0.456 | 0.208 | -4.235 | -2.446 | -7.321 | 0.000 |
| Young-middle | Haskell-Ramsay et al. 2017 | Young-middle | -2.718 | 0.490 | 0.240 | -3.679 | -1.757 | -5.543 | 0.000 |
| Young-middle | Karabay et al. 2018(Low dose) | Young-middle | -0.395 | 0.206 | 0.042 | -0.799 | 0.009 | -1.917 | 0.055 |
| Young-middle | Karabay et al. 2018(High dose) | Young-middle | -0.163 | 0.204 | 0.042 | -0.564 | 0.238 | -0.797 | 0.425 |
| Young-middle | | | -0.894 | 0.314 | 0.099 | -1.510 | -0.279 | -2.850 | 0.004 |

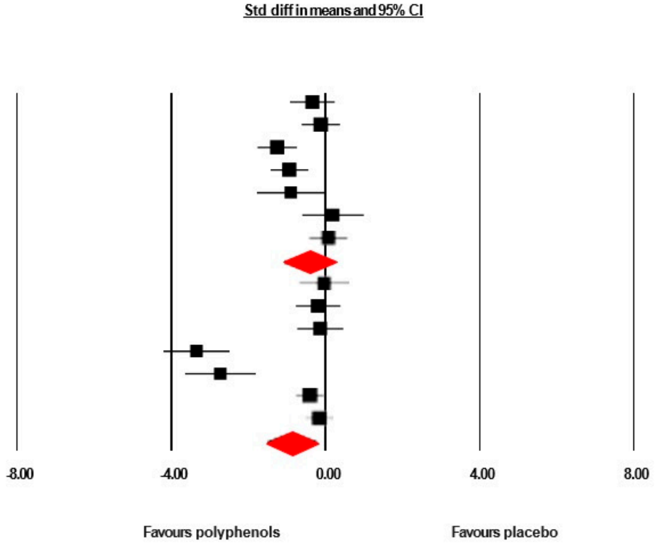


**Figure S1.** Plot of studies investigating the effect of (poly)phenols-rich supplementation on psychomotor function in old- and young/middle-aged adults.

| Group by subgroup within study | Study name | Intervention duration | Statistics for each study | | | | | | |
|---|---|---|---|---|---|---|---|---|---|
| | | | Std diff in means | Standard error | Variance | Lower limit | Upper limit | Z-Value | p-Value |
| Acute | Francis et al. 2006 | Acute | -0.051 | 0.354 | 0.125 | -0.744 | 0.642 | -0.143 | 0.886 |
| Acute | Massee et al. 2015 | Acute | -0.192 | 0.317 | 0.100 | -0.813 | 0.429 | -0.605 | 0.545 |
| Acute | Dietz et al. 2017 | Acute | -3.341 | 0.456 | 0.208 | -4.235 | -2.446 | -7.321 | 0.000 |
| Acute | Haskell-Ramsay et al. 2017 | Acute | -2.718 | 0.490 | 0.240 | -3.679 | -1.757 | -5.543 | 0.000 |
| Acute | Karabay et al. 2018(Low dose) | Acute | -0.395 | 0.206 | 0.042 | -0.799 | 0.009 | -1.917 | 0.055 |
| Acute | Karabay et al. 2018(High dose) | Acute | -0.163 | 0.204 | 0.042 | -0.564 | 0.238 | -0.797 | 0.425 |
| Acute | | | -1.023 | 0.339 | 0.115 | -1.688 | -0.359 | -3.018 | 0.003 |
| Chronic | Mix & Crews. 2000 | Chronic | -0.335 | 0.318 | 0.101 | -0.959 | 0.290 | -1.051 | 0.293 |
| Chronic | Kritz-Silverstein et al. 2003 | Chronic | -0.120 | 0.275 | 0.076 | -0.659 | 0.419 | -0.437 | 0.662 |
| Chronic | Mastroiacovo et al. 2015 (High dose) | Chronic | -1.247 | 0.282 | 0.080 | -1.800 | -0.694 | -4.418 | 0.000 |
| Chronic | Mastroiacovo et al. 2015 (Medium dose) | Chronic | -0.933 | 0.272 | 0.074 | -1.466 | -0.400 | -3.431 | 0.001 |
| Chronic | Massee et al. 2015 | Chronic | -0.134 | 0.325 | 0.106 | -0.771 | 0.502 | -0.413 | 0.680 |
| Chronic | Antomet al. 2018 (High dose) | Chronic | -0.895 | 0.469 | 0.220 | -1.815 | 0.024 | -1.909 | 0.056 |
| Chronic | Antomet al. 2018 (Low dose) | Chronic | 0.192 | 0.429 | 0.184 | -0.649 | 1.033 | 0.448 | 0.655 |
| Chronic | Huhn et al. 2018 | Chronic | 0.065 | 0.275 | 0.076 | -0.474 | 0.603 | 0.236 | 0.814 |
| Chronic | | | -0.428 | 0.292 | 0.085 | -1.000 | 0.144 | -1.465 | 0.143 |

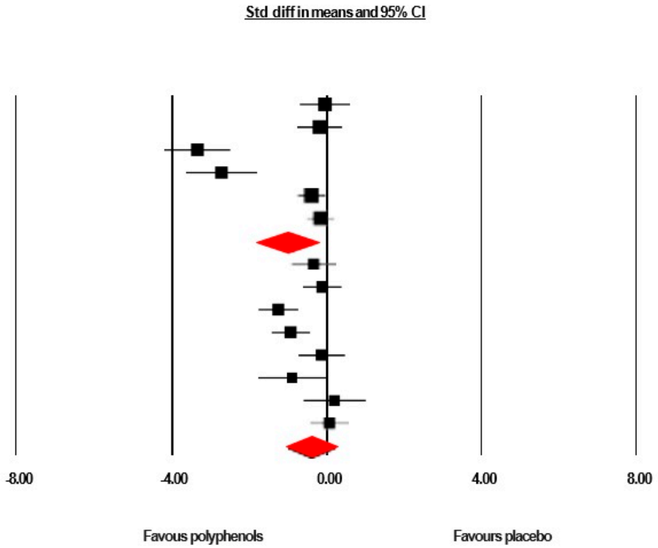


**Figure S2.** Plot of studies investigating the acute and chronic effect of (poly) phenols-rich supplementation on psychomotor function.

| Group by subgroup within study | Study name | Human bioavailability | Statistics for each study | | | | | | | Std diff in means and 95% CI |
|---|---|---|---|---|---|---|---|---|---|---|
| | | | Stddiff in means | Standard error | Variance | Lower limit | Upper limit | Z-Value | p-Value | |
| Low | Mix & Crews, 2000 | Low | -0.335 | 0.318 | 0.101 | -0.959 | 0.290 | -1.051 | 0.293 | |
| Low | Haskell-Ramsay et al. 2017 | Low | -2.718 | 0.490 | 0.240 | -3.679 | -1.757 | -5.543 | 0.000 | |
| Low | Antomet al. 2018 (High dose) | Low | -0.895 | 0.469 | 0.220 | -1.815 | 0.024 | -1.909 | 0.056 | |
| Low | Antomet al. 2018 (Low dose) | Low | 0.192 | 0.429 | 0.184 | -0.649 | 1.033 | 0.448 | 0.655 | |
| Low | Huhn et al. 2018c | Low | 0.065 | 0.275 | 0.076 | -0.474 | 0.603 | 0.236 | 0.814 | |
| Low | | | -0.678 | 0.392 | 0.154 | -1.446 | 0.091 | -1.728 | 0.084 | |
| Medium | Francis et al. 2006 | Medium | -0.051 | 0.354 | 0.125 | -0.744 | 0.642 | -0.143 | 0.886 | |
| Medium | Mastroiacovo et al. 2015 (High dose) | Medium | -1.247 | 0.282 | 0.080 | -1.800 | -0.694 | -4.418 | 0.000 | |
| Medium | Mastroiacovo et al. 2015 (Medium dose) | Medium | -0.933 | 0.272 | 0.074 | -1.466 | -0.400 | -3.431 | 0.001 | |
| Medium | Massee et al. 2015 (Acute) | Medium | -0.192 | 0.317 | 0.100 | -0.813 | 0.429 | -0.605 | 0.545 | |
| Medium | Massee et al. 2015 (Chronic) | Medium | -0.134 | 0.325 | 0.106 | -0.771 | 0.502 | -0.413 | 0.680 | |
| Medium | Dietz et al. 2017 (Acute) | Medium | -3.341 | 0.456 | 0.208 | -4.235 | -2.446 | -7.321 | 0.000 | |
| Medium | Karabay et al. 2018 (Low dose) | Medium | -0.395 | 0.206 | 0.042 | -0.799 | 0.009 | -1.917 | 0.055 | |
| Medium | Karabay et al. 2018 (High dose) | Medium | -0.163 | 0.204 | 0.042 | -0.564 | 0.238 | -0.797 | 0.425 | |
| Medium | | | -0.760 | 0.297 | 0.088 | -1.341 | -0.178 | -2.562 | 0.010 | |

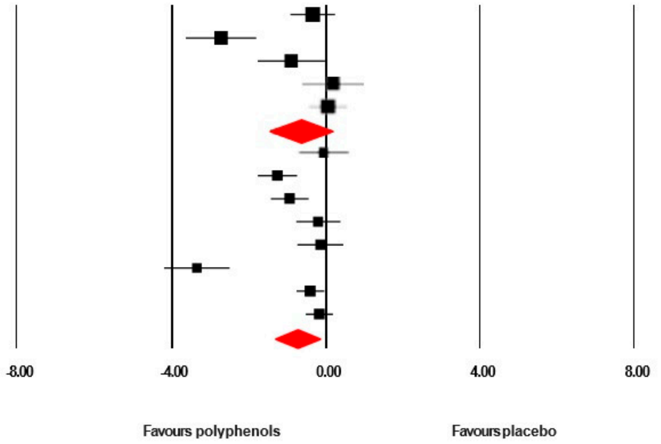


**Figure S3.** Plot of studies investigating the effect of (poly) phenols-rich supplementation with low and medium human bioavailability on psychomotor function.

| Study name | Cumulative statistics | | | | | | |
| --- | --- | --- | --- | --- | --- | --- | --- |
| | Point | Standard error | Variance | Lower limit | Upper limit | Z-Value | p-Value |
| Mix & Crews, 2000 | -0.335 | 0.318 | 0.101 | -0.959 | 0.290 | -1.051 | 0.293 |
| Kritz-Silverstein et al. 2003 | -0.212 | 0.208 | 0.043 | -0.620 | 0.196 | -1.018 | 0.309 |
| Francis et al. 2006 | -0.170 | 0.179 | 0.032 | -0.522 | 0.181 | -0.950 | 0.342 |
| Mastroiacovo et al. 2015 (High dose) | -0.451 | 0.289 | 0.084 | -1.018 | 0.116 | -1.560 | 0.119 |
| Mastroiacovo et al. 2015 (Medium dose) | -0.555 | 0.239 | 0.057 | -1.024 | -0.086 | -2.320 | 0.020 |
| Massee et al. 2015 (Acute) | -0.498 | 0.207 | 0.043 | -0.904 | -0.092 | -2.405 | 0.016 |
| Massee et al. 2015 (Chronic) | -0.450 | 0.185 | 0.034 | -0.812 | -0.088 | -2.435 | 0.015 |
| Dietz et al. 2017 | -0.756 | 0.307 | 0.094 | -1.357 | -0.155 | -2.467 | 0.014 |
| Haskell-Ramsay et al. 2017 | -0.955 | 0.323 | 0.104 | -1.589 | -0.322 | -2.957 | 0.003 |
| Antom et al. 2018 (High dose) | -0.947 | 0.298 | 0.089 | -1.531 | -0.363 | -3.179 | 0.001 |
| Antom et al. 2018 (Low dose) | -0.849 | 0.283 | 0.080 | -1.404 | -0.293 | -2.994 | 0.003 |
| Huhn et al. 2018 | -0.766 | 0.265 | 0.070 | -1.284 | -0.247 | -2.895 | 0.004 |
| Karabay et al. 2018 (Low dose) | -0.727 | 0.233 | 0.054 | -1.184 | -0.271 | -3.124 | 0.002 |
| Karabay et al. 2018 (High dose) | -0.677 | 0.211 | 0.045 | -1.090 | -0.263 | -3.208 | 0.001 |
| | -0.677 | 0.211 | 0.045 | -1.090 | -0.263 | -3.208 | 0.001 |

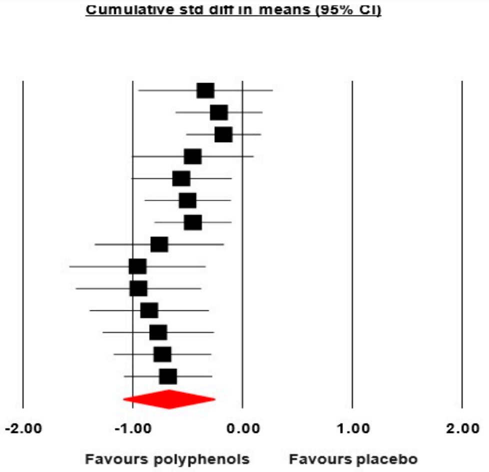


| Study name | Statistics with study removed | | | | | | |
| --- | --- | --- | --- | --- | --- | --- | --- |
| | Point | Standard error | Variance | Lower limit | Upper limit | Z-Value | p-Value |
| Mix & Crews, 2000 | -0.707 | 0.227 | 0.051 | -1.152 | -0.263 | -3.118 | 0.002 |
| Kritz-Silverstein et al. 2003 | -0.726 | 0.227 | 0.052 | -1.171 | -0.281 | -3.198 | 0.001 |
| Francis et al. 2006 | -0.726 | 0.223 | 0.050 | -1.164 | -0.289 | -3.252 | 0.001 |
| Mastroiacovo et al. 2015 (High dose) | -0.630 | 0.219 | 0.048 | -1.060 | -0.200 | -2.872 | 0.004 |
| Mastroiacovo et al. 2015 (Medium dose) | -0.659 | 0.227 | 0.051 | -1.103 | -0.215 | -2.908 | 0.004 |
| Massee et al. 2015 (Acute) | -0.718 | 0.226 | 0.051 | -1.161 | -0.275 | -3.179 | 0.001 |
| Massee et al. 2015 (Chronic) | -0.722 | 0.225 | 0.051 | -1.163 | -0.281 | -3.208 | 0.001 |
| Dietz et al. 2017 | -0.483 | 0.164 | 0.027 | -0.804 | -0.162 | -2.946 | 0.003 |
| Haskell-Ramsay et al. 2017 | -0.541 | 0.193 | 0.037 | -0.920 | -0.162 | -2.797 | 0.005 |
| Antom et al. 2018 (High dose) | -0.664 | 0.221 | 0.049 | -1.098 | -0.231 | -3.002 | 0.003 |
| Antom et al. 2018 (Low dose) | -0.737 | 0.220 | 0.048 | -1.168 | -0.307 | -3.357 | 0.001 |
| Huhn et al. 2018 | -0.740 | 0.224 | 0.050 | -1.178 | -0.301 | -3.308 | 0.001 |
| Karabay et al. 2018 (Low dose) | -0.709 | 0.237 | 0.056 | -1.173 | -0.245 | -2.994 | 0.003 |
| Karabay et al. 2018 (High dose) | -0.727 | 0.233 | 0.054 | -1.184 | -0.271 | -3.124 | 0.002 |
| | -0.677 | 0.211 | 0.045 | -1.090 | -0.263 | -3.208 | 0.001 |

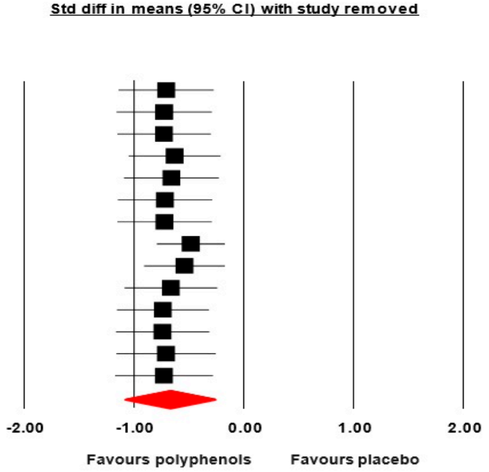


**Figure S4.** Cumulative statistics and statistics with study removed for studies investigating the effect of (poly)phenols-rich supplementation on psychomotor functions.

| Group by subgroup within study | Study name | Std diff in means | Subgroup within study | Standard error | Variance | Lower limit | Upper limit | Z-Value | p-Value |
|---|---|---|---|---|---|---|---|---|---|
| Older | Witte et al. 2014 | -0.161 | Older | 0.295 | 0.087 | -0.739 | 0.418 | -0.544 | 0.587 |
| Older | Bowtell et al. 2017 | 0.411 | Older | 0.397 | 0.158 | -0.368 | 1.190 | 1.033 | 0.301 |
| Older | Huhn et al. 2018 | -0.004 | Older | 0.275 | 0.075 | -0.543 | 0.534 | -0.016 | 0.987 |
| Older | | 0.073 | | 0.536 | 0.287 | -0.977 | 1.123 | 0.136 | 0.892 |
| Young-middle | Decroix et al. 2016 | 3.148 | Young-middle | 0.611 | 0.373 | 1.951 | 4.346 | 5.154 | 0.000 |
| Young-middle | Sadowska-Krepa et al. 2017 | 4.442 | Young-middle | 0.878 | 0.770 | 2.722 | 6.162 | 5.061 | 0.000 |
| Young-middle | Sadowska-Krepa et al. 2019 | 0.400 | Young-middle | 0.505 | 0.255 | -0.590 | 1.389 | 0.791 | 0.429 |
| Young-middle | | 2.409 | | 0.629 | 0.395 | 1.176 | 3.641 | 3.831 | 0.000 |

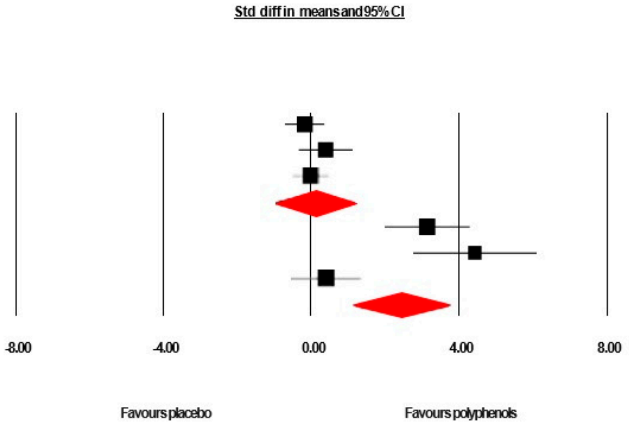


**Figure S5.** Plot of studies investigating the effect of (poly) phenols-rich supplementation on BDNF in old- and young/middle-aged adults.

| Group by subgroup within study | Study name | Std diff in means | Subgroup within study | Standard error | Variance | Lower limit | Upper limit | Z-Value | p-Value |
|---|---|---|---|---|---|---|---|---|---|
| Low | Witte et al. 2014 | -0.161 | Low | 0.295 | 0.087 | -0.739 | 0.418 | -0.544 | 0.587 |
| Low | Bowtell et al. 2017 | 0.411 | Low | 0.397 | 0.158 | -0.368 | 1.190 | 1.033 | 0.301 |
| Low | Huhn et al. 2018 | -0.004 | Low | 0.275 | 0.075 | -0.543 | 0.534 | -0.016 | 0.987 |
| Low | Sadowska-Krepa et al. 2019 | 0.400 | Low | 0.505 | 0.255 | -0.590 | 1.389 | 0.791 | 0.429 |
| Low | | 0.065 | | 0.169 | 0.029 | -0.267 | 0.396 | 0.383 | 0.701 |
| Medium | Decroix et al. 2016 | 3.148 | Medium | 0.611 | 0.373 | 1.951 | 4.346 | 5.154 | 0.000 |
| Medium | Sadowska-Krepa et al. 2017 | 4.442 | Medium | 0.878 | 0.770 | 2.722 | 6.162 | 5.061 | 0.000 |
| Medium | | 3.570 | | 0.501 | 0.251 | 2.588 | 4.553 | 7.121 | 0.000 |

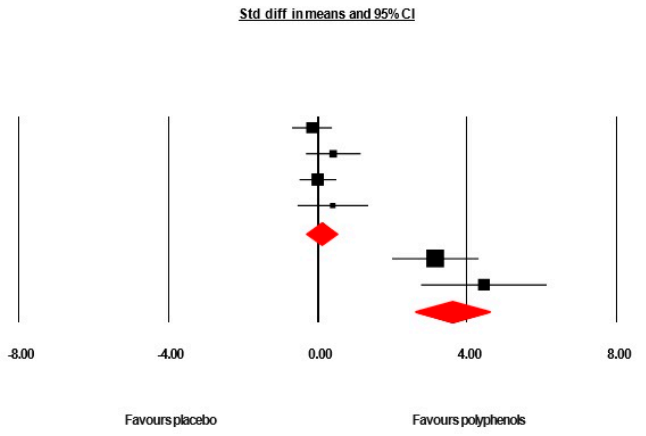


**Figure S6.** Plot of studies investigating the effect of (poly)phenols-rich supplementation with low and medium human bioavailability on BDNF.

| Study name | Cumulative statistics | | | | | | | Cumulative std diff in means (95% CI) |
|---|---|---|---|---|---|---|---|---|
| | Point | Standard error | Variance | Lower limit | Upper limit | Z-Value | p-Value | |
| Witte et al. 2014 | -0.161 | 0.295 | 0.087 | -0.739 | 0.418 | -0.544 | 0.587 | |
| Decroix et al. 2016 | 1.451 | 1.654 | 2.735 | -1.791 | 4.692 | 0.877 | 0.380 | |
| Bowtell et al. 2017 | 1.051 | 0.828 | 0.685 | -0.571 | 2.673 | 1.269 | 0.204 | |
| Sadowska-Krepa et al. 2017 | 1.821 | 0.910 | 0.828 | 0.037 | 3.604 | 2.001 | 0.045 | |
| Huhn et al. 2018 | 1.356 | 0.633 | 0.400 | 0.116 | 2.596 | 2.143 | 0.032 | |
| Sadowska-Krepa et al. 2019 | 1.168 | 0.531 | 0.282 | 0.127 | 2.209 | 2.199 | 0.028 | |
| | 1.168 | 0.531 | 0.282 | 0.127 | 2.209 | 2.199 | 0.028 | |

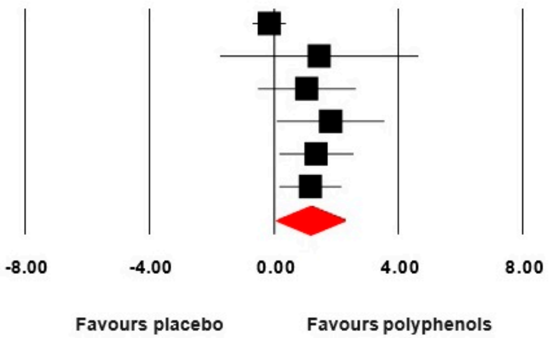


| Study name | Statistics with study removed | | | | | | | STD diff in means (95% CI) with study removed |
|---|---|---|---|---|---|---|---|---|
| | Point | Standard error | Variance | Lower limit | Upper limit | Z-Value | p-Value | |
| Witte et al. 2014 | 1.519 | 0.680 | 0.462 | 0.186 | 2.851 | 2.233 | 0.026 | |
| Decroix et al. 2016 (Acute) | 0.712 | 0.459 | 0.211 | -0.188 | 1.612 | 1.549 | 0.121 | |
| Bowtell et al. 2017 | 1.379 | 0.665 | 0.442 | 0.076 | 2.682 | 2.075 | 0.038 | |
| Sadowska-Krepa et al. 2017 (Chronic) | 0.643 | 0.436 | 0.190 | -0.211 | 1.498 | 1.476 | 0.140 | |
| Huhn et al. 2018 | 1.497 | 0.706 | 0.499 | 0.112 | 2.881 | 2.119 | 0.034 | |
| Sadowska-Krepa et al. 2019 (Chronic) | 1.356 | 0.633 | 0.400 | 0.116 | 2.596 | 2.143 | 0.032 | |
| | 1.168 | 0.531 | 0.282 | 0.127 | 2.209 | 2.199 | 0.028 | |

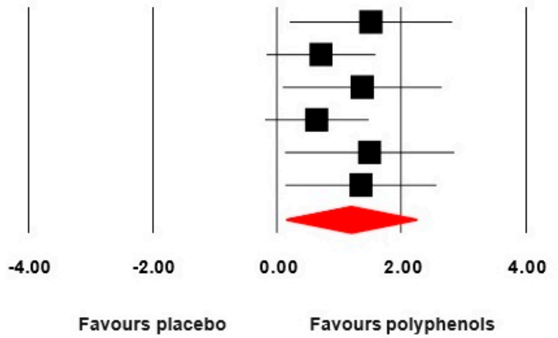


**Figure S7.** Cumulative statistics and statistics with study removed for studies investigating the effect of (poly)phenols-rich supplementation on BDNF.
